# Supplementary material for: Role of CD4/CD8 ratio on the incidence of tuberculosis in HIV-infected patients on antiretroviral therapy followed up for more than a decade
Source: PLoS One. 2020 May 22;15(5):e0233049. doi: 10.1371/journal.pone.0233049 (PMC7244128; doi:10.1371/journal.pone.0233049)
Supplement: S1 Table — (DOCX) [file pone.0233049.s001.docx]

**Supporting Table 1. Incidence of tuberculosis in HIV-infected patients on antiretroviral treatment followed for up to 15 years**

| **Years of ART follow-up** | **No. of incident TB cases (%)** | **Person years at risk** | **Incidence rate per 100 person year follow-up (95% CI)** |
| --- | --- | --- | --- |
| **In all patients (n=347)** | | | |
| **Overall** | 47 (100.0) | 3258.72 | 1.44 (1.08 – 1.92) |
| **< 5** | 36 (76.6) | 67.89 | 53.03 (38.25 – 73.51) |
| **5 - 10** | 8 (17.0) | 489.66 | 1.63 (0.82 – 3.27) |
| **>10** | 3 (6.4) | 2701.17 | 0.11 (0.04 – 0.34) |
| **Virally suppressed patients only (n=233)** | | | |
| **Overall** | 28 (100) | 2492.06 | 1.12 (0.78 – 1.63) |
| **< 5** | 22 (78.6) | 33.39 | 65.89 (43.39 – 100.07) |
| **5 - 10** | 3 (10.7) | 311.50 | 0.96 (0.31 – 2.99) |
| **>10** | 3 (10.7) | 2147.17 | 0.14 (0.05 – 0.43) |
