# Supplementary material for: Role of CD4/CD8 ratio on the incidence of tuberculosis in HIV-infected patients on antiretroviral therapy followed up for more than a decade
Source: PLoS One. 2020 May 22;15(5):e0233049. doi: 10.1371/journal.pone.0233049 (PMC7244128; doi:10.1371/journal.pone.0233049)
Supplement: S2 Table — (DOCX) [file pone.0233049.s002.docx]

**Supplemental Table 2: Incidence rate of tuberculosis vs. updated CD4/CD8 category.**

| **CD4/CD8 category** | **All patients** | **Virological outcome** | |
| --- | --- | --- | --- |
|  |  | **Suppressed** | **Not suppressed** |
| < 0.30 | 6.05 (4.28 – 8.55)* | 5.41 (3.32 – 8.83) | 5.05 (2.52 – 10.09) |
| 0.30 – 0.45 | 0.85 (0.35 – 2.05) | 0.87 (0.33 – 2.31) | 1.50 (0.21 – 10.66) |
| > 0.45 | 0.47 (0.25 – 0.87) | 0.46 (0.23 – 0.92) | 1.62 (0.40 – 6.47) |

***data are incidence rate (IQR)**
