# Supplementary figures and images for: Role of CD4/CD8 ratio on the incidence of tuberculosis in HIV-infected patients on antiretroviral therapy followed up for more than a decade
Source: PLoS One. 2020 May 22;15(5):e0233049. doi: 10.1371/journal.pone.0233049 (PMC7244128; doi:10.1371/journal.pone.0233049)

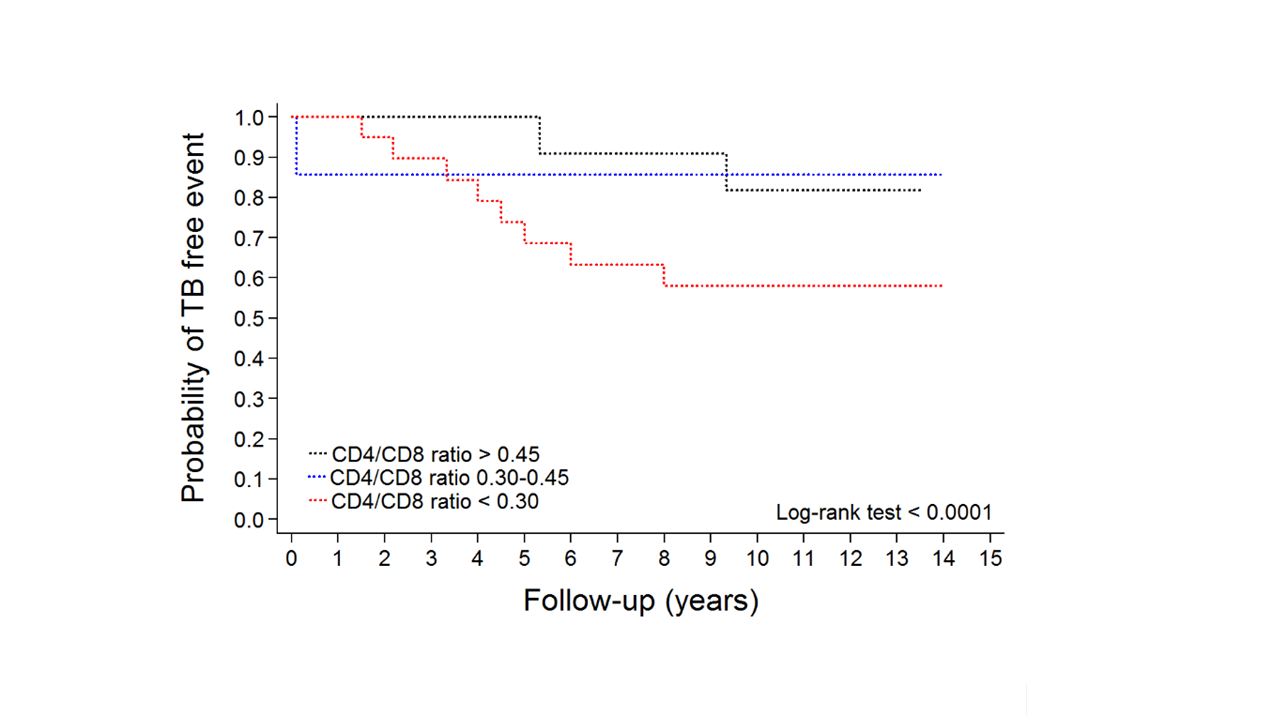

Supplement: S1 Fig — Data are from those with virological failure. (TIF) [file pone.0233049.s003.tif]
