# Supplementary material for: Role of CD4/CD8 ratio on the incidence of tuberculosis in HIV-infected patients on antiretroviral therapy followed up for more than a decade
Source: PLoS One. 2020 May 22;15(5):e0233049. doi: 10.1371/journal.pone.0233049 (PMC7244128; doi:10.1371/journal.pone.0233049)
Supplement: Attachment — Submitted filename: Response to Reviewers.docx [file pone.0233049.s004.docx]

**Response to Reviewers**

**PONE-D-19-29695** Role of CD4/CD8 ratio on the incidence of tuberculosis in HIV-infected patients on antiretroviral therapy followed up for more than a decade

We thank the academic editor and reviewers for their critical evaluation and their constructive criticisms.

We provide the following response to the comments raised:

Academic editor:

- We agree with the comment that we used CD4/CD8 ratio data available around the time of incident TB. We have now included an additional analysis (see revised figure 2) showing longitudinal data in the cohort who developed incident TB vs. those who did not develop TB. The results suggest that though patients who developed TB respond to ART, the response is indeed smaller and suboptimal as compared to the responses noted among the patients who did not develop TB.

Major comments:

Reviewer #1:

- The diagnosis of TB was based on the national guidelines. We have added a reference for this in the revised manuscript. The majority of the patients in our cohort indeed presented with extrapulmonary TB (72%), which is expected in HIV-infected patients. Diagnosis for such cases is confirmed by pathology. In addition, the challenge related to the diagnosis of TB in sub-Saharan Africa has been noted as a shortcoming of the study in the discussion section of the manuscript. We do have also included similar references from Ethiopia as well as other countries in Africa.

Reviewer #2:

1. We have added evidence from our longitudinal data showing that persistently low CD4/CD8 ratio overtime is a risk factor for developing incident TB (revised figure 2).
2. We agree with the reviewer that IPT impacts on development of TB. We have provided references for this in the original manuscript too. Our database lacks information with respect to IPT adherence. This shortcoming is now noted in the discussion section of the revised manuscript.
3. Data for those who failed to achieve viral suppression has also been included (Figure 1 and Figure3).

Minor comment:

- That the data collected was retrospective has been noted both in the abstract and the methods sections.
